# Characterization of a novel AA3_1 xylooligosaccharide dehydrogenase from *Thermothelomyces myriococcoides* CBS 398.93

**DOI:** 10.1186/s13068-022-02231-w

**Published:** 2022-12-07

**Authors:** Hongbo Zhao, Johanna Karppi, Thi Truc Minh Nguyen, Annie Bellemare, Adrian Tsang, Emma Master, Maija Tenkanen

**Affiliations:** 1 Department of Food and Nutrition, University of Helsinki, Helsinki, Finland; 2 Department of Bioproducts and Biosystems, Aalto University, Espoo, Finland; 3 Centre for Structural and Functional Genomics, Concordia University, 7141 Sherbrooke Street West, Montreal, QC H4B 1R6 Canada; 4 Department of Chemical Engineering and Applied Chemistry, University of Toronto, Toronto, ON Canada

**Keywords:** Xylooligosaccharide dehydrogenase, Cellobiose dehydrogenase, *Thermothelomyces myriococcoides*, CAZy AA3, AA3_1

## Abstract

**Background:**

The Carbohydrate-Active enZymes (CAZy) auxiliary activity family 3 (AA3) comprises flavin adenine dinucleotide-dependent (FAD) oxidoreductases from the glucose–methanol–choline (GMC) family, which play auxiliary roles in lignocellulose conversion. The AA3 subfamily 1 predominantly consists of cellobiose dehydrogenases (CDHs) that typically comprise a dehydrogenase domain, a cytochrome domain, and a carbohydrate-binding module from family 1 (CBM1).

**Results:**

In this work, an AA3_1 gene from *T. myriococcoides* CBS 398.93 encoding only a GMC dehydrogenase domain was expressed in *Aspergillus niger*. Like previously characterized CDHs, this enzyme (*Tm*XdhA) predominantly accepts linear saccharides with β-(1 → 4) linkage and targets the hydroxyl on the reducing anomeric carbon. *Tm*XdhA was distinguished, however, by its preferential activity towards xylooligosaccharides over cellooligosaccharides. Amino acid sequence analysis showed that *Tm*XdhA possesses a glutamine at the substrate-binding site rather than a threonine or serine that occupies this position in previously characterized CDHs, and structural models suggest the glutamine in *Tm*XdhA could facilitate binding to pentose sugars.

**Conclusions:**

The biochemical analysis of *Tm*XdhA revealed a catalytic preference for xylooligosaccharide substrates. The modeled structure of *Tm*XdhA provides a reference for the screening of oxidoreductases targeting xylooligosaccharides. We anticipate *Tm*XdhA to be a good candidate for the conversion of xylooligosaccharides to added-value chemicals by its exceptional catalytic ability.

**Supplementary Information:**

The online version contains supplementary material available at 10.1186/s13068-022-02231-w.

## Background

Lignocellulosic biomass is the most abundantly available biomaterial on earth and is considered an essential resource for the production of biofuels and chemicals. Lignocellulosic biomass is mainly composed of plant cell wall materials including cellulose, hemicelluloses and lignin, which can be degraded by a cocktail of multiple enzymes secreted by fungi and other microbes. Such enzymes are classified in the Carbohydrate-Active enZymes (CAZy) database; among them, the redox enzymes showing auxiliary roles in lignocellulose degradation are classified in the “Auxiliary Activities” (AA) families [1]. AA families comprising carbohydrate-active enzymes include cellobiose dehydrogenases (EC 1.1.99.18, cellobiose: acceptor 1-oxidoreductase, AA3_1), glucose oxidases (EC 1.1.3.4, AA3_2), pyranose dehydrogenases (EC 1.1.99.29, AA3_2), pyranose 2-oxidases (EC 1.1.3.10, AA3_4), galactose oxidases (EC 1.1.3.9, AA5_2), oligosaccharide oxidases (EC 1.1.3.-, AA7), and lytic polysaccharide monooxygenases (LPMOs, ECs 1.14.99.-, 1.14.99.53-.56, from families AA9-11 and AA13-16).

The AA3 family consists of glucose–methanol–choline (GMC) oxidoreductases with varying auxiliary activities that are divided into four subfamilies. The subfamily AA3_1 mainly consists of CDHs, which were first discovered in the 1970s in the white-rot fungi *Trametes versicolor* [2] and *Phanerochaete chrysosporium* P127-1 [3]. The CDHs accept a wide spectrum of electron acceptors including one-electron acceptors like cytochrome *c*, ferric-ion complexes, Cu^2+^; two-electron acceptors like dichlorophenolindophenol (DCIP), benzoquinone (BQ), molecular oxygen, and enzymatic electron acceptors such as lytic polysaccharide monooxygenase (LPMO) [4, 5]. CDHs have been shown to specifically oxidize the anomeric carbon forming the corresponding lactone through a reductive half-reaction mechanism [6]. The lactone then spontaneously hydrolyses to carboxylic acid in water. CDHs characterized to date generally show preferential activity towards β-(1 → 4)-linked cellobiose and lactose, which differ from cellobiose by the orientation of the C-4 hydroxyl at the non-reducing end [7]. CDHs from *Sclerotium (Athelia) rolfsii* CBS 191.62 (*Sr*CDH)*, Trametes pubescens* MB 89 (*Tp*CDH)*, Trametes villosa* MB 51 (*Tv*CDH)*, Humicola insolens* (*Hi*CDH)*, and Neurospora crassa* CBS 232.56 (*Nc*CDHs) also act on xylooligosaccharides, including xylobiose and xylotriose, albeit with comparatively low catalytic efficiency relative to cellobiose [8–11]. Other oligosaccharides, including maltose, maltotriose, maltotetraose, galactobiose, mannobiose, and various monosaccharides can also be accepted by some CDHs with relatively low affinity [12].

CDHs typically comprise a flavin adenine dinucleotide (FAD) binding GMC dehydrogenase domain, an N-terminal cytochrome domain, and in some cases a C-terminal family 1 cellulose binding module (CBM1) [4]. Four classes of CDHs were identified based on the phylogenetic analysis, with class-I CDHs from basidiomycetes and the classes II, III, IV from ascomycetes with very few exceptions [13]. Depending on the presence of a CBM, the class-II CDHs are further divided into class-IIA harboring a CBM1 and class-IIB without CBM1 [14]. The cytochrome domain of CDHs is normally connected to the GMC dehydrogenase domain by a papain-sensitive amino acid linker. In some cases, the cytochrome domain can serve as a redox mediator that activates LPMOs for oxidative cellulose depolymerization [5, 15]. However, it appears that the cytochrome domain is not strictly necessary for the physiological function of all CDHs since CDHs bearing only the dehydrogenase domain are observed in all four phylogenetic classes [13].

*Thermothelomyces myriococcoides* CBS 389.93 (syn. *Crassicarpon hotsonii, and Myriococcum thermophilum*) is an ascomycete which was originated from the surface of heated compost in Switzerland [16, 17]. It encodes 17 AA3s, a typical number for fungal organisms, among which four genes are predicted to encode enzymes belonging to family AA3_1 (Gene accession code: *Myrth2p4_000359*, *Myrth2p4_001304*, *Myrth2p4_007444*, and *Myrth2p4_005287*) [18]. The *Myrth2p4_000359* gene encodes only the dehydrogenase domain, and to our knowledge, no such native protein has been functionally characterized [13]. Previous studies have shown that after the proteolytic cleave of the cytochrome, the remaining CDH retains activity [11, 19, 20]. In this study, we heterologously produced the Myrth2p4_000359 protein for detailed functional characterization. The enzyme was found to preferentially oxidize xylooligosaccharides over cellobiose and other cellooligosaccharides, which has not been seen for previously characterized AA3_1s. Based on the nomenclature proposed by [21], this enzyme is named as xylooligosaccharide dehydrogenase (*Tm*XdhA) and its corresponding gene as *TmxdhA.*

## Results

### Selection of gene *Myrth2p4_000359* and production of *Tm*XdhA

**Fig. 1:**
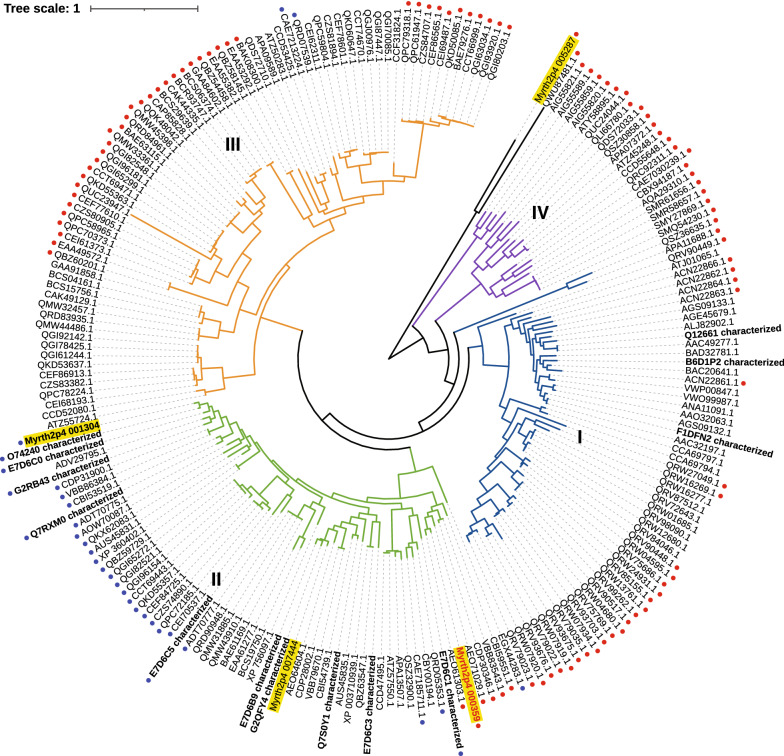
Phylogenetic tree built from dehydrogenase domain of AA3_1 proteins collected from CAZy and four AA3_1 proteins from *T. myriococcoides* CBS 389.93. Characterized proteins from CAZy are in bold font and the four proteins from *T. myriococcoides* are highlighted in yellow. *Tm*XdhA characterized in this study is marked with red font. Blue dots indicate proteins with CBM1 domain and red dots indicate proteins without cytochrome domain. The details of the previously characterized AA3_1s can be found in Additional file 1

The gene models of the genome of *Thermothelomyces myriococcoides* CBS 389.93 (*Crassicarpon hotsonii, Myriococcum thermophilum*) were functionally annotated using multiple tools and databases including InterProScan, blastp search of the CAZy and SwissProt databases, and HMMs from dbCAN database. The *T. myriococcoides* genome harbors genes predicted to encode 4 enzymes from family AA1 (laccases); 4 AA2s (peroxidases); 18 AA3s (flavoenzymes including four AA3_1s); 1 AA5_1 (glyoxal oxidase); 17 AA7s (oligosaccharide oxidases), and 24 LPMOs from family AA9, 3 LPMOs from family AA11, and 1 LPMO from family AA13. Three *T. myriococcoides* AA3_1 sequences (*Myrth2p4_000359*, *Myrth2p4_001304* and *Myrth2p4_007444*) were classified as class-II and the fourth (*Myrth2p4_005287*) is phylogenetically distinct with other three AA3_1 genes encoded by *T. myriococcoides* (Fig. 1). CDH encoded by *Myrth2p4_001304* (*Mt*CDH, Uniprot: A9XK88) has been characterized previously. *Mt*CDH comprises a cytochrome, GMC dehydrogenase and CBM1 domains, and was shown to mainly oxidize cellooligosaccharides and lactose [5, 22–25]. From the other two Class-II sequences, *Myrth2p4_007444* contains both cytochrome and GMC dehydrogenase domains while *Myrth2p4_000359* contains only the GMC dehydrogenase domain (Table 1). Herein, *Myrth2p4_000359 (TmxdhA)* was selected for recombinant production and functional characterization.

**Table 1:** Amino acid sequence length and domain architectures of the three class-II AA3_1 proteins from *T. myriococcoides*

Class	Gene accession code	Protein	Amino acid length	Domain architectures
IIB	*Myrth2p4_000359*	*Tm*XdhA	578	
IIA	*Myrth2p4_001304*	*Mt*CDH	828	
IIB	*Myrth2p4_007444*	Uncharacterized	787	

*DH* GMC dehydrogenase domain, *Cyt* cytochrome domain, *CBM* carbohydrate-binding module

### Production of *Tm*XdhA

*Tm*XdhA was heterologously produced in *A. niger* and the recombinant production generated a glycosylated protein with electrophoretic molecular weight of approximately 140 kDa, which is nearly double that of the theoretical molecular weight (62.2 kDa) (Additional file 2: Fig. S1A). A 2D-electrophoretic analysis for the deglycosylated protein at the denatured condition revealed two separate spots (Additional file 2: Fig. S1B). Spot one had a molecular weight of about 95 kDa and a p*I* approximately at 4.6 while spot two showed a protein with a molecular weight of about 85 kDa and a p*I* approximately at 5.2 (Additional file 2: Fig. S1B). MALDI-TOF-MS analysis of tryptic peptides confirmed the production of *Tm*XdhA (spot 2), as well as presence of a predicted hydrolase endogenous to *A. niger* (spot 1, UniProt: A0A370CB09). Substantial efforts to remove the hydrolase from the *Tm*XdhA preparation were not successful. Accordingly, the hydrolytic activity was tested using five different *p*NP-glycosides; whereas no to negligible activity was detected on *p*NP-β-d-glucopyranoside, *p*NP-β-d-xylopyranoside and *p*NP-β-d-mannopyranoside, activity was detected on *p*NP-α-l-arabinofuranoside (0.52 U/mg) and *p*NP-α-d-glucopyranoside (0.12 U/mg) (Additional file 2: Table S1). The α-glucosidase activity was therefore minimized through the addition of 0.1 mM castanospermine, which did not influence the oxidoreductase activity (Additional file 2: Table S1); the low side α-arabinofuranosidase activity, however, remained.

### *Tm*XdhA oxidizes a variety of mono-, di- and oligosaccharides at the anomeric carbon

**Fig. 2:**
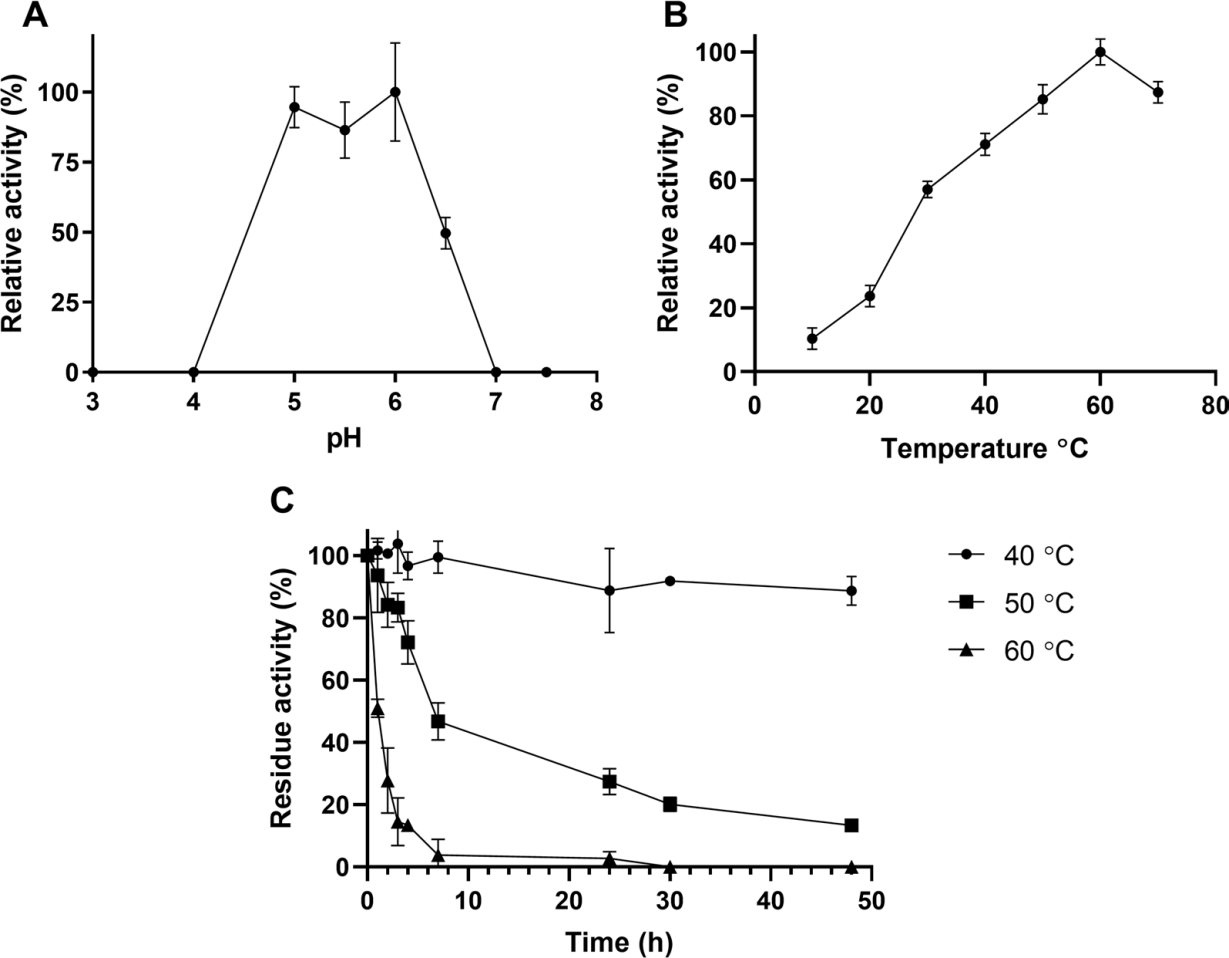
*Tm*XdhA activity at different pH values at 30 °C (**A**), temperatures at pH 5.5 (**B**), and residual activity after incubation at different temperatures at pH 5.5 (**C**). Activity was measured on cellobiose with DCIP as the electron acceptor. Error bars represent standard deviation of three replicate reactions

Maximum *Tm*XdhA activity towards cellobiose, using DCIP as the electron acceptor, was measured between pH 5.0 and 6.0 (Fig. 2A), which is similar to the pH preference reported for *Mt*CDH [25]. The highest enzyme activity at pH 5.5 was observed at 60 °C (Fig. 2B) and the half-life of *Tm*XdhA activity at 60 °C was 1 h (Fig. 2C). The half-life at 50 °C was 7 h and *Tm*XdhA was stable (residue activity > 90%) at 40 °C for no less than 48 h.

Cellobiose oxidation by *Tm*XdhA was more efficient when using BQ (9.6 ± 0.70 U/mg) rather than DCIP (5.3 ± 1.10 U/mg) as the electron acceptor. No oxidase activity of *Tm*XdhA was detected in the coupled reaction with horseradish peroxidase and ABTS tracking the formation of hydrogen peroxide. However, *Tm*XdhA was found to oxidize cellobiose to a small extent in a 24-h reaction without the presence of BQ and DCIP, indicating that *Tm*XdhA is a weak oxidase (data not shown). Based on these findings, *Tm*XdhA activity towards additional substrates was tested in 10 mM ammonium acetate buffer (pH 5.5) using BQ as the electron acceptor in 24 h reactions.

*Tm*XdhA oxidized 18 of the 29 tested substrates, albeit it to varying degrees (Table 2). *Tm*XdhA oxidation of glucose, galactose and mannose, reached nearly 90% after 24 h. Xylose itself could not be detected by ESI-Q-TOF-MS possibly due to the low ionization capacity of the method. Instead, *Tm*XdhA oxidation of xylose generated xylonic acid in the deprotonated form was observed as *m/z* 165. Besides monosaccharides, the complete oxidation of β-(1 → 4)-linked disaccharides cellobiose, xylobiose and lactose was observed; whereas β-(1 → 4)-linked mannobiose and glucosyl-mannose were oxidized to 50% and 90%, respectively (Table 2). Trace oxidation (< 5%) of α-(1 → 4)-linked maltose was observed; however, β-(1 → 4)-linked galactobiose and chitosanbiose as well as glucodisaccharides with α/β-(1 → 2)-, α/β-(1 → 3)-, α/β-(1 → 6)-glucosidic bonds were not accepted as substrates by *Tm*XdhA.

**Table 2:** Kinetic parameters of *Tm*XdhA for cellooligosaccharides and xylooligosaccharides

Substrate	*K*_M_ (mM)	*k*_cat_ (s^−1^)	*k*_cat_*/K*_M_ (s^−1^∙mM^−1^)
Cellobiose	0.36 ± 0.04	16.7 ± 0.7	46.2 ± 5.8
Cellotriose	0.43 ± 0.04	17.6 ± 0.6	40.1 ± 4.2
Cellotetraose	0.53 ± 0.04	23.8 ± 1.1	45.1 ± 4.0
Xylobiose	0.16 ± 0.01	17.5 ± 0.4	108.9 ± 9.5
Xylotriose	0.11 ± 0.01	22.3 ± 0.4	200.9 ± 14.5
Xylotetraose	0.13 ± 0.01	25.0 ± 0.5	187.7 ± 15.2

Standard deviation is calculated from three replicate reactions

Longer cello- and xylo-oligosaccharides, including cellotriose, cellotetraose, xylotriose, xylotetraose, and acidic oligosaccharide 2^3^-(4-O-methyl-α-d-glucuronyl)-xylotriose (U^4m2^XX) were fully oxidized by *Tm*XdhA; *Tm*XdhA also partially (80%) oxidized 3^2^-β-d-glucosyl-cellobiose after 24 h. By contrast, oxidation of 2^2^-(4-*O*-methyl-α-d-glucuronyl)-xylobiose (U^4m2^X) was not detected. When testing *Tm*XdhA activity on arabinofuranosyl (Ara*f*) substituted xylooligosaccharides, 3^2^-α-l-arabinofuranosyl-xylobiose (A^3^X) and 2^3^-α-l-arabinofuranosyl-xylotriose (A^2^XX), the residual α-arabinofuranosidase activity completely hydrolysed A^3^X and A^2^XX during the long 24-h treatment. During shorter 3-h incubation, *Tm*XdhA was found to only oxidize A^2^XX and no oxidation of A^3^X was detected (Additional file 2: Fig. S2). *Tm*XdhA did not show any activity on C-1 methylated cellobiose and lactose.

Because the oxidized products were analyzed by ESI-Q-TOF-MS in negative mode, oxidation at the reducing anomeric center would be negatively charged and detected in their anionic form [M-H]^−^; while neutral substrates would be detected as chlorine adducts [M + Cl]^−^. Thus, oxidation at the anomeric center is equivalent to a 20 Dalton (Da) decrease relative to the substrate. Herein, the tested hexoses with mass-to-charge ratio (*m/z*) of 215 generated products with *m/z* of 195, consistent with oxidation at the reducing anomeric center (Table 2). Similarly, a loss of *m/z* 20 was detected for all oxidized neutral substrates. The acidic substrates U^4m2^X and U^4m2^XX carrying Methyl-α-D-Glucuronyl group were detected as deprotonized forms [M–H]^−^ with peaks at *m/z* 471 and *m/z* 603, respectively. The oxidation of U^4m2^XX introduced a second carboxylic acid at the reducing end, hence, the oxidized U^4m2^XX can be both single charged [M–H]^−^ and doubly charged [M–2H]^2^ and was detected as peaks at *m/z* 619 and *m/z* 309, respectively.

**Fig. 3:**
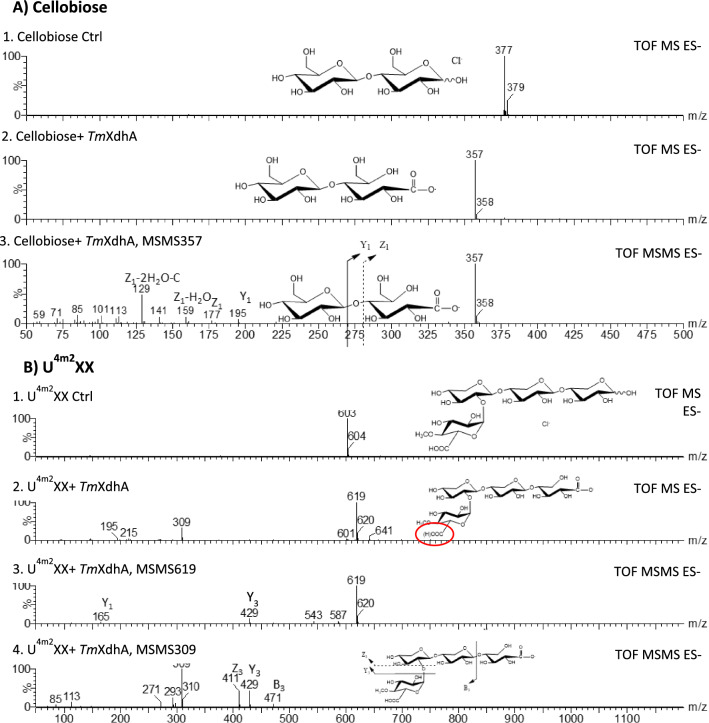
MS and MS/MS spectra collected in negative ion mode showing the ionization and fragmentation of cellobiose (**A**) and 2^3^-(4-*O*-methyl-α-D-glucuronyl)-xylotriose (U^4m2^XX) (**B**) after oxidation by *Tm*XdhA. **A1**. Negative MS spectra of solely cellobiose, **A2**. Negative MS spectra of the reaction products from *Tm*XdhA cellobiose reaction, and **A3**. MS/MS 357 of *Tm*XdhA oxidized cellobiose. **B1**. MS spectra of U^4m2^XX, **B2**. MS spectra of *Tm*XdhA U^4m2^XX reaction products, **B3**. MS/MS 619 of *Tm*XdhA oxidized U^4m2^XX, and **B4**. MS/MS 309 of *Tm*XdhA oxidized U^4m2^XX

Fragmentation of oxidized oligosaccharides by ESI-Q-TOF-MS/MS confirmed that the substrates were oxidized solely at their anomeric center (Fig. 3). Results from cellobiose and U^4m2^XX are shown as examples. With cellobiose (Fig. 3A), Y-ion and Z-ions from glycosidic bond cleavage were the most abundant fragment ions. The fragmentation for the oxidized U^4m2^XX resulted Y-ion, Z-ion, and B-ions (Fig. 3B). In both cases, the molecular masses for Y_1_- and Z_1_-ions increased by 16 Da, compared to the unoxidized control sample, supporting that the oxidation reaction occurred in the reducing glycoside and the formation of C-1 carboxylic acid.

### *Tm*XdhA prefers xylooligosaccharide substrates

**Fig. 4:**
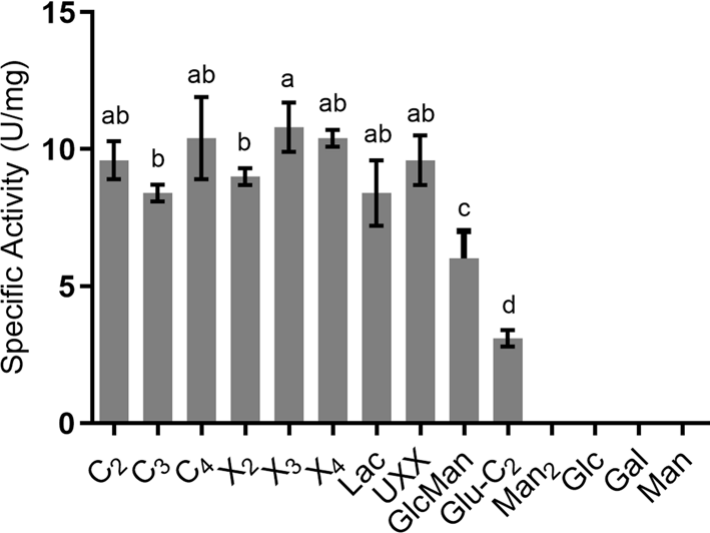
Specific activity of *Tm*XdhA towards different substrates (*n* = 3). No activity was detected for mannobiose (Man_2_), glucose (Glc), galactose (Gal), and mannose (Man). Error bars represent standard deviation of three replicate reactions. Significance in activities (*p* < 0.05) is shown by different lowercase letters (a-d). Cellobiose, C_2_; cellotriose, C_3_; cellotetraose, C_4_; xylobiose, X_2_; xylotriose, X_3_; xylotetraose, X_4_; lactose, Lac; 2^3^-(4-*O*-methyl-α-d-glucuronyl)-xylotriose, UXX; Glucosyl-(1 → 4)-β-D-mannose, GlcMan Glucosyl-(1 → 3)-β-d-cellobiose, Glc-C_2_

*Tm*XdhA oxidized the cellooligosaccharides and xylooligosaccharides with the specific activities between 8.4 and 11 U/mg (Fig. 4). The specific activity for *Tm*XdhA towards xylotriose (10.8 ± 0.9 U/mg) is statistically higher than the specific activity towards xylobiose (9.0 ± 0.3 U/mg) and cellotriose (8.4 ± 0.3 U/mg); whereas activity towards xylotriose was not significantly different from activities on cellobiose and cellotetraose (9.6 ± 0.7 U/mg and 10.4 ± 1.5 U/mg, respectively). *Tm*XdhA oxidized lactose (8.4 ± 1.2 U/mg) and U^4m2^XX (9.6 ± 0.9 U/mg) in the similar level compared to the cello- and xylo-oligosaccharides; while significantly lower activity was found towards glucosyl-(1 → 3)-β-d-cellobiose (3.1 ± 0.3 U/mg) and glucosyl-(1 → 4)-β-d-mannose (6.0 ± 1.0 U/mg). Though mannobiose, glucose, galactose, and mannose were oxidized in the 24 h reaction, no quantifiable activity was detected towards mannobiose, glucose, galactose, and mannose during the initial velocity measurement.

Kinetic analyses revealed the high catalytic efficiency of *Tm*XdhA towards xylooligosaccharides, mainly driven by the comparatively low *K*_M_ of *Tm*XdhA towards the xylooligosaccharide substrates (Table 3). Overall, *Tm*XdhA had higher binding affinity for xylooligosaccharides than cellooligosaccharides, with 3–5 times smaller *K*_M_ values on the xylooligosaccharides than for the cellooligosaccharides. Increased values in *k*_cat_ were observed with the increasing length of both cello- and xylo-oligosaccharides, and the *K*_M_ values for oxidation of cellooligosaccharides followed the same pattern. On the contrary, the highest affinity of *Tm*XdhA was towards xylotriose (0.11 mM) compared to xylobiose (0.16 mM) and xylotetraose (0.13 mM). Notably, the catalytic efficiency (*k*_cat_/*K*_M_) showed that xylotriose and xylotetraose are the most favorable and, cellotriose the least favorable of the tested substrates.

**Table 3:** Oxidation of mono-, di- and oligosaccharides by *Tm*XdhA after 24 h with product detection by ESI-Q-TOF-MS in the negative mode

	Substrate	Linkage	Oxidation	Substrate	Oxidized product	Degree of oxidation
				*m/z*	*m/z*	%
Monosaccharides	Glucose		Y	215	195	~ 90
	Xylose		Y	^b^	165	^b^
	Mannose		Y	215	195	~ 90
	Galactose		Y	215	195	~ 90
Disaccharides	Cellobiose	β 1 → 4	Y	377	357	100
	Sophorose	β 1 → 2	N	377		–
	Laminaribiose	β 1 → 3	N	377		–
	Gentiobiose	β 1 → 6	N	377		–
	Maltose^a^	α 1 → 4	Y (Trace)	377	357	< 5
	Kojibiose^a^	α 1 → 2	N	377		–
	Nigerose^a^	α 1 → 3	N	377		–
	Isomaltose^a^	α 1 → 6	N	377		–
	Xylobiose	β 1 → 4	Y	317	297	100
	Lactose	β 1 → 4	Y	377	357	100
	Galactobiose	β 1 → 4	N	377		–
	Mannobiose	β 1 → 4	Y	377	357	~ 50
	Glucosyl-mannose	β 1 → 4	Y	377	357	~ 90
	Chitosanbiose	β 1 → 4	N	375		–
Oligosaccharides	Cellotriose	β 1 → 4	Y	539	519	100
	Cellotetraose	β 1 → 4	Y	701	681	100
	Glucosyl-(1 → 3)-β-D-cellobiose	β 1 → 3/4	Y	539	519	~ 80
	Xylotriose	β 1 → 4	Y	449	429	100
	Xylotetraose	β 1 → 4	Y	581	561	100
Acidic	U^4m2^X	β 1 → 4; α 1 → 2	N	471		–
	U^4m2^XX	β 1 → 4; α 1 → 2	Y	603	619, 309	100
C-1 methylated disaccharides	Methylated-cellobiose	β 1 → 4	N	391		0
	Methylated-lactose	β 1 → 4	N	391		0

Detected oxidation is indicated as Y and no oxidation as N. Degree of oxidation was calculated with Eq. 1. All substrates except for 2^2^-(4-*O*-methyl-α-d-glucuronyl)-xylobiose (U^4m2^X) and 2^3^-(4-*O*-methyl-α-d-glucuronyl)-xylotriose (U^4m2^XX) were detected as chlorine adducts [M + Cl]^−^ while U^4m2^X and U^4m2^XX were detected in their anionic form [M–H]^−^ and [M–2H]^2−^. The degree of oxidation was estimated using Eq. 1 in Material and method section

^a^Castanospermine included in the reactions

^b^Xylose does not give a clear peak; hence, the percentage was not calculable

**Fig. 5:**
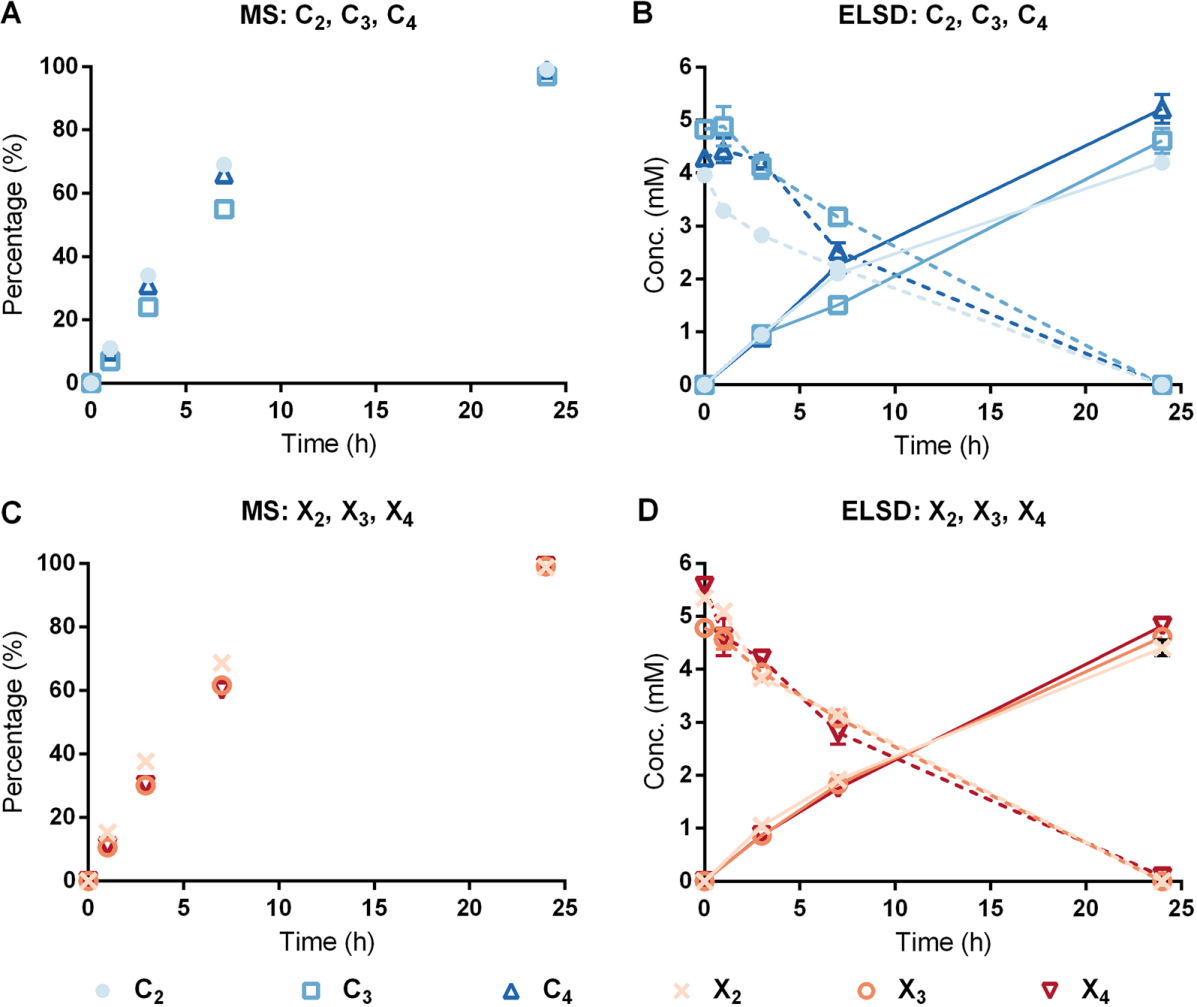
Product formation and substrate depletion by *Tm*XdhA. Reactions with cellobiose (C_2_), cellotriose (C_3_), and cellotetraose (C_4_) followed with MS (**A**) and with HILIC-ELSD (**B**). Reactions with xylobiose (X_2_), xylotriose (X_3_), and xylotetraose (X_4_) followed with MS (**C**) and with HILIC-ELSD (**D**). HILIC-ELSD results show the decrease of the substrate with dashed line and formation of the reaction product with continuous line. Error bars represent standard deviation of three replicate reactions

Consistent with the kinetic analysis, product formation followed by both quantitative HILIC-ELSD and semi-quantitative MS method indicated little impact of degree of polymerization on *Tm*XdhA activity towards cellooligosaccharides and xylooligosaccharides (Fig. 5). Overall, all tested oligosaccharides were found to be oxidized in similar speed and be fully converted to corresponding aldonic acids after 24 h. The HILIC-ELSD method to track the formation of oxidized carbohydrates is time-consuming and requires complex pre-treatment of reaction mixtures prior to their analysis. Semi-quantitative MS method was applied here as an alternative method for the identification and semi-quantification of the oxidized carbohydrates. The MS method is simpler and faster than HILIC-ELSD, however it slightly overestimates the formed products compared to HILIC-ELSD. The difference in percentage were most apparent at the 7-h time point, where the conversion for cello-series was 30–50% and for xylo-series was 40–50% detected with HILIC-ELSD and the corresponding oxidation percentage calculated based on MS data were 55–70% and 60–70%, respectively (Fig. 5). Based on this comparison, the results from HILIC-ELSD are included in the Additional file 2 (Fig. S5) for the following reactions and only the results from the MS method are discussed further.

**Fig. 6:**
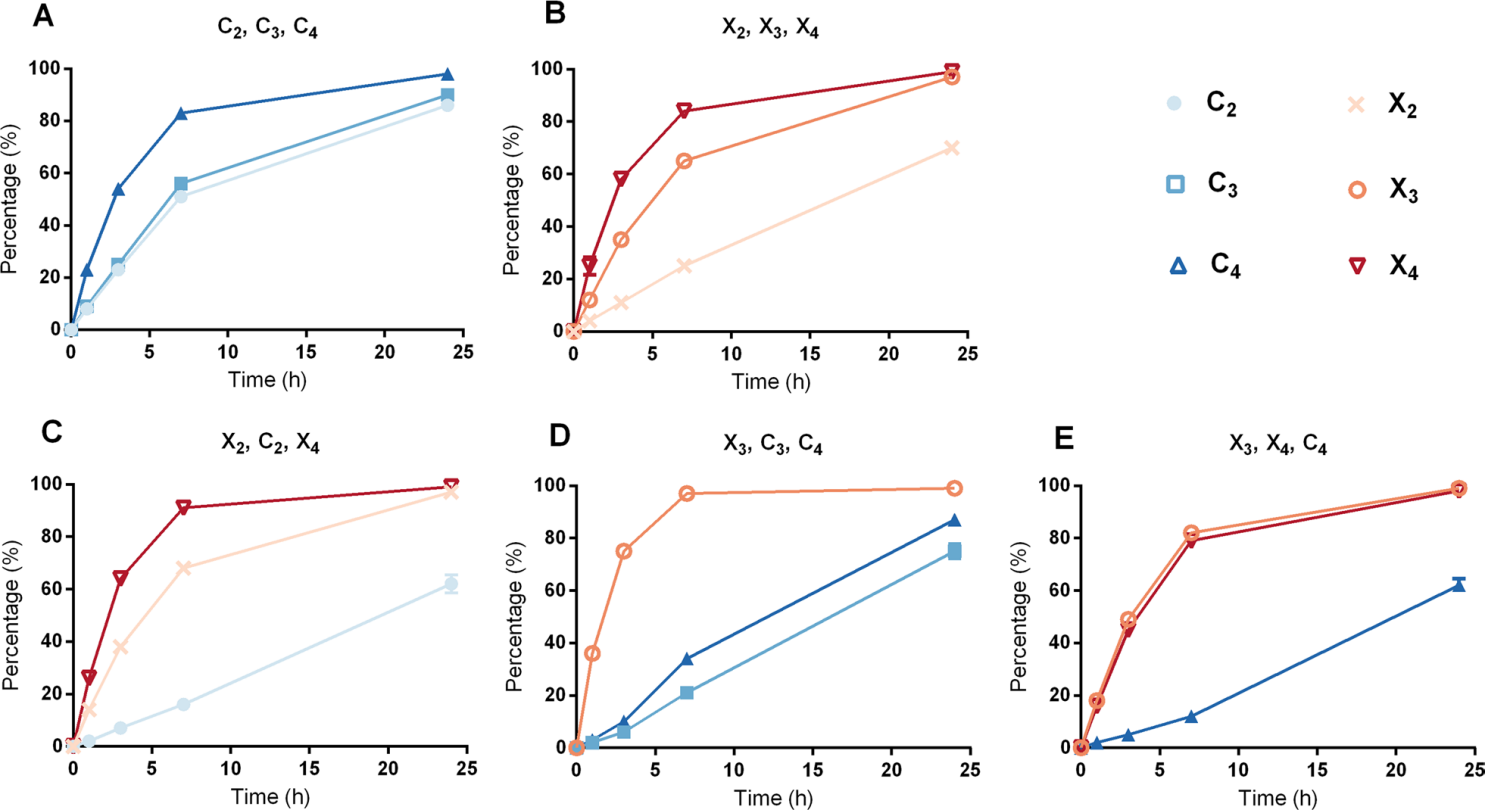
Product formation during *Tm*XdhA oxidation of cellooligosaccharide and xylooligosaccharide mixtures followed by the MS method. Reactions contained following oligosaccharides as substrates: cellobiose (C_2_), cellotriose (C_3_), and cellotetraose (C_4_). (**A**); xylobiose (X_2_), xylotriose (X_3_), and xylotetraose (X_4_) (**B**); xylobiose, cellobiose, and xylotetraose (**C**); xylotriose, cellotriose, and cellotetraose (**D**); and xylotriose, xylotetraose, and cellotetraose (**E**). Error bars represent standard deviation of three replicate reactions

The impact of degree of polymerization was revealed when *Tm*XdhA activity was tested on separate mixtures of xylooligosaccharides and cellooligosaccharides (Fig. 6). In this case, cellotetraose and xylotetraose were oxidized faster compared to the corresponding shorter oligosaccharides (Fig. 6A, B). Still, when testing *Tm*XdhA on oligosaccharide mixtures comprising both xylooligosaccharides and cellooligosaccharides, *Tm*XdhA preference towards xylooligosaccharides over the cellooligosaccharides was observed regardless of the degree of polymerization (Fig. 6C–E).

### Structural features of the *Tm*XdhA model

**Fig. 7:**
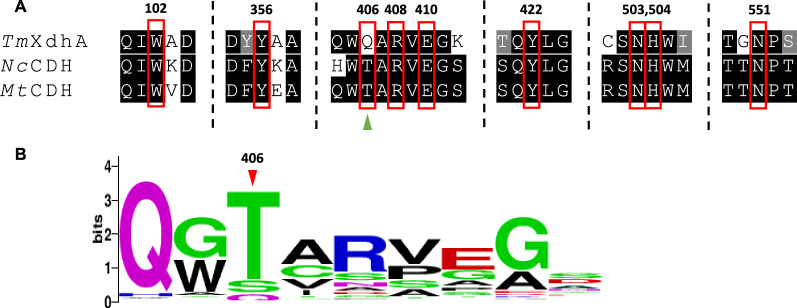
**A** Alignment of *Tm*XdhA with two structurally characterized CDHs. *Nc*CDH: cellobiose dehydrogenase from *N. crassa* (PDB code 4QI7); *Mt*CDH: cellobiose dehydrogenase from *T. myriococcoides* (PDB code 4QI4, strain CBS 208.89). The catalytically important amino acids are highlighted by red boxes. The only difference is highlighted with green triangle. **B** Sequence logos of the residues around 406Q from all CAZy AA3_1 sequences and *Tm*XdhA

Using Basic Local Alignment Search Tool (BLAST) analysis, the *Tm*XdhA sequence shared the highest sequence identity to two structurally and biochemically characterized CDHs, *Mt*CDH from the same organism *T. myriococcoides* CBS 208.89 (PDB code 4QI4) and a CDH from *Neurospora crassa* CBS 232.56 (*Nc*CDH, PDB code 4QI7), 63% and 62%, respectively. By aligning the sequences of *Tm*XdhA and all other CAZy AA3_1s, especially the key residues involved in the substrate binding and catalysis defined in [5], a glutamine residue is found at position 406, other than the threonine residue at the same place for *Mt*CDH and *Nc*CDH (Fig. 7A). The position 406 is found to be conserved with polar amino acid residues, including threonine, serine, and glutamine, with glutamine as the least abundant one within all CAZy AA3_1 sequences (Fig. 7B).

**Fig. 8:**
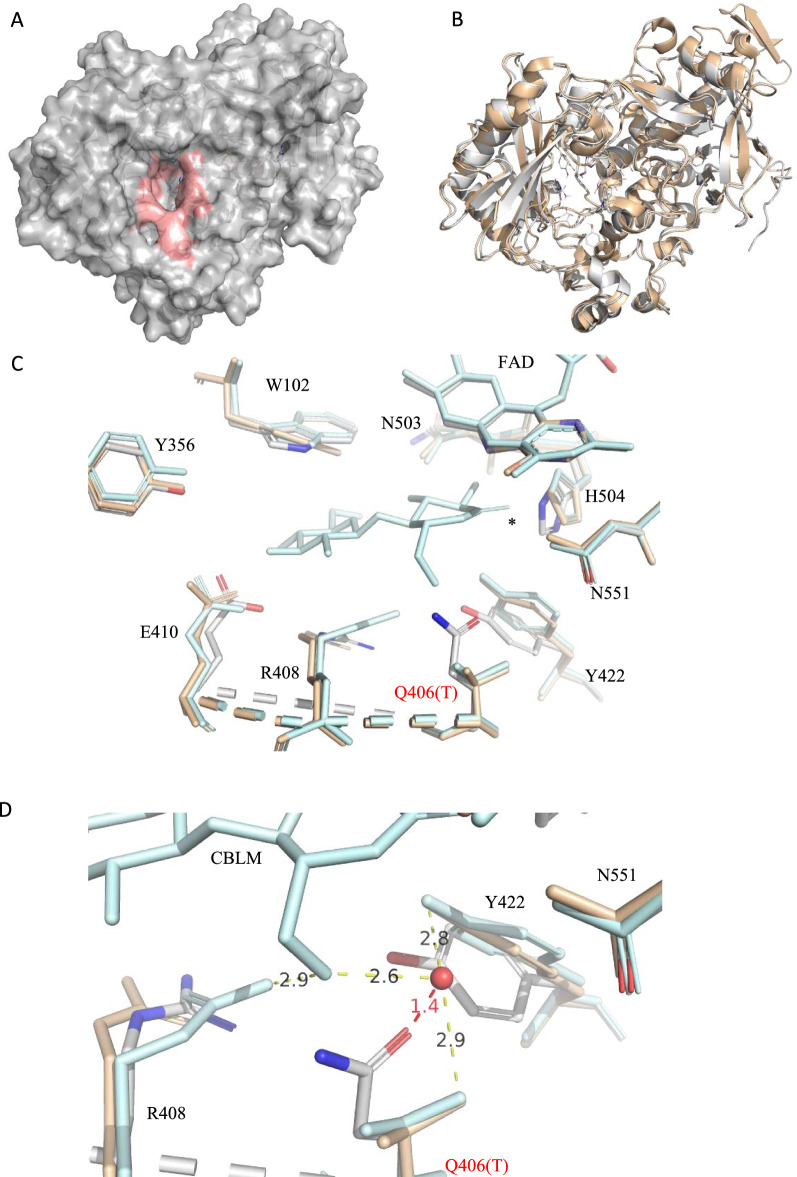
Structural comparison of *Tm*XdhA predicted model and *Mt*CDH (PDB code 4QI4). **A** The predicted structure of *Tm*XdhA created with AlphaFold2, the surface of active site is colored in red. **B** Superimposition of *Tm*XdhA predicted model structure with structure of the dehydrogenase domain of *Mt*CDH (PDB code 4QI4), the structure of *Mt*CDH is shown in light yellow color and the structure of *Tm*XdhA is shown in gray. **C** Alignment for the active site of *Tm*XdhA (white), substrate free *Mt*CDH (yellow), and *Mt*CDH-cellobionolactam (Blue, PDB code 4QI5). The asterisk marks the position that corresponds to the hydroxyl being abstracted by CDHs in cellobiose. **D** Alignment for the reducing end moiety C-6 binding site of the *Tm*XdhA (white) and *Mt*CDH -CBLM complex (blue). The water molecular mediating the hydrogen bond (yellow dash line) system is shown as red sphere. The distance between the OE1 of Q406 and the water molecule is shown as red dash line

In an effort to predict sequence and structural determinants of AA3_1 activity towards xylooligosaccharides, the structure prediction of *Tm*XdhA was created using AlphaFold2, which is suggested to be a highly accurate protein structure predictor [26]. Most of the residues were modeled at with the per-residue confidence (pLDDT) score higher than 90 and the predicted aligned error (PAE) lower than 5 (Additional file 2: Fig. S6). The predicted *Tm*XdhA structure possesses an architecture with an active-site pocket and it folds in the same way as the *Mt*CDH structure, while *Mt*CDH has an extra CBM domain at the C-terminus (Fig. 8A, B). The catalytic histidine residue is located at position 504 for *Tm*XdhA that is considered to be the proton acceptor according to the alignment. Previous results have shown that the polar threonine residue in *Mt*CDH forms a water-mediated hydrogen bond systems that hold the C-6 of the reducing motif of cellobionolactam (CBLM) in its position [5]. Instead, *Tm*XdhA possesses a longer glutamine residue (Q406) at the same position, which limits the space between the water molecule and OE1 of Q406 to only 1.4 Å.

## Discussion

CAZy AA3_1 enzymes, mainly CDHs, have been extensively studied over the past decades and have the potential to be used in wide variety of applications including biosensors, biomedical applications, functionalization of partially degraded cellulose and hemicelluloses, and enhancing lignocellulose degradation [12, 19, 27–29].

Many fungi possess paralogous genes that could express multiple isoenzymes within the same family or subfamily. Such feature is called multigenicity. In the four subfamilies of AA3s, multigenicity is most observed in AA3_2 subfamily and least observed in the AA3_1 subfamily [30]. The isoenzymes from AA3_2 could differ completely in their transcription and secretion level [11, 31], and may vary in their ability to utilize different electron acceptors and substrates [32, 33]. So far investigated kinetic parameter support the multigenicity of *T. myriococcoides* AA3_1 proteins to assist in adapting habitat with diverse substrate availability. *Mt*CDH exhibits ten times lower *K*_M_ towards cellobiose, cellotriose and cellotetraose (0.03–0.06 mM) compared to *Tm*XdhA (0.36–0.53 mM) [23]. Although *Mt*CDH reportedly oxidized xylooligosaccharides (DP1-6), kinetic parameters for the *Mt*CDH on xylooligosaccharides have not been published [22].

Consistent with the previously characterized AA3_1 CDHs, the β-(1 → 4)-linkage resulting the linear substrate structure is important for substrate acceptance by *Tm*XdhA [7]. Though the activity is only towards reducing end C-1, *Tm*XdhA activity is diminished on trisaccharide substrates where the non-reducing neutral or acidic sugar residue links to penultimate residue via linkages other than β-(1 → 4) (e.g., as in the case of A^3^X, U^4m2^X, and glucosyl-β-(1 → 3)-cellobiose). Similar to *Mt*CDH and *Pc*CDH from *P. chrysosporium* K3, the equatorial position of the C-4 hydroxyl group and unoccupied equatorial position of the C-2 hydroxyl group are important for *Tm*XdhA activity [5, 6].

AA3_1 enzymes clearly vary in their ability to oxidize xylooligosaccharides. For instance, the *Pc*CDH from Basidiomycota was found to primarily accept cellobiose, but could not oxidize xylobiose, xylotriose, or Glc-Glc-Xyl, Xyl-Glc-Xyl, and Glc-Xyl-Xyl [34], while xylobiose was oxidized by *Nc*CDHs and *Hi*CDHs from Ascomycota, the catalytic efficiency was 50 to 2500 times lower than that towards cellobiose [10, 11]. By contrast, the affinity and the catalytic efficiency for *Tm*XdhA towards xylobiose, xylotriose and xylotetraose was more than ten times higher compared to all previous characterized CDHs [12]. The preferential xylooligosaccharide oxidizing feature of *Tm*XdhA is unique compared to other characterized AA3_1 CDHs. Some C1 acting AA7 oligosaccharide oxidases have also been reported to act on xylooligosaccharides and the AA7 oxidase from *Myceliophthora thermophile* C1 was observed to exhibits a strong substrate preference toward xylooligosaccharides. However, this AA7 xylooligosaccharide oxidase has ten times lower catalytic efficiency towards xylooligosaccharides compared to *Tm*XdhA [35]. Instead, the AA7 oligosaccharide oxidase from *Sarocladium strictum* T1 possess catalytic efficiency in the same magnitude towards xylooligosaccharides as *Tm*XdhA, but does not have a preference towards xylooligosaccharides and oxidizes cellooligosaccharide with similar efficiency [36]. This feature of *Tm*XdhA can be used in biosensors for lower detection limit of xylooligosaccharides, generate xylan-based aldonic acid, and selective removal of xylan fractions from lignocellulosic materials.

*Tm*XdhA differs with *Mt*CDH and *Nc*CDH in one residue in the substrate-binding domain on sequence level (T406/Q406). The threonine for *Mt*CDH will generate a hydrogen bond system for the locking of the C-6 of the reducing end moiety into the substrate-binding site [5]. However, based on the *Tm*XdhA model, the space between the water molecule and OE1 of Q406 in *Tm*XdhA is limited (1.4 Å). Hence, a new hydrogen bond system to promote the binding towards pento-pyranosidic moiety is expected. These results open up possibility to find new xylooligosaccharide active enzymes from the uncharacterized CAZymes and to engineer CDHs for better xylooligosaccharide oxidation performance. The predicted model of *Tm*XdhA has similar structure compared to other CDHs and glucose oxidases, with a conserved histidine (H504) residue positioned next to the flavin ring. Such structure facilitates the reductive half-reaction, where the histidine abstracts the proton from the hydroxyl on the anomeric carbon (C-1) while the hydrogen from C-1 transfers to the FAD as hydride [6]. Our attempt failed in redirecting the *Tm*XdhA oxidation to positions other than reducing end C-1 blocking the reducing end by methylation.

In our study, the *Tm*XdhA had better affinity and catalytic efficiency towards higher oligosaccharides and oxidized xylotriose and xylotetraose three to six times quicker than xylobiose in oligosaccharide mixtures, suggesting novel binding site within the catalytic domain for the third xylosidic moiety. The kinetic parameters do not always follow to the real substrate preference in an oligosaccharide mixture as shown in our study. Giving xylooligosaccharides as an example, the *K*_M_ increased by 50% from cellobiose to cellotetraose while the catalytic efficiency was about the same for cellobiose, cellotriose and cellotetraose. Nevertheless, we found that cellotetraose is the preferred substrate compared to cellobiose and cellotriose in a cellooligosaccharide mixture. Such inconsistency could be caused by the inhibition effect of the formed aldonic acids.

*Tm*XdhA represents a unique type of AA3_1 protein containing only a dehydrogenase domain, it lacks the cytochrome and CBM domains of other characterized AA3_1 members [11, 19, 20]. Analyses of AA3_1 sequences have shown *cdhs* lacking cytochrome domain and CBM, yet no such native enzyme has been functionally characterized. The direct electron transfer from CDH to LPMO was previously shown to be mediated by the cytochrome domain of some CDHs [5]. More recently, the FAD domain of *Mt*CDH was found to mediate electron transfer to an LPMO [20], consistent with the potential of other single domain flavoenzymes such as AA3_2 glucose dehydrogenase, AA3_2 aryl alcohol oxidases, and AA7 oligosaccharide oxidases to mediate electron transfer to LPMOs [37, 38]. Hence, it would be worth testing in the future how single domain enzyme *Tm*XdhA cooperate with LPMOs.

## Conclusions

In summary, this work recombinantly expressed and biochemically characterized a dehydrogenase domain only AA3_1 enzyme (*Tm*XdhA) for the first time. *Tm*XdhA was found to have unique preferential activity towards xylooligosaccharides, which is the first xylooligosaccharide dehydrogenase discovered in the AA3_1 family. Additionally, *Tm*XdhA oxidized longer oligosaccharides quicker than disaccharides in oligosaccharide mixtures, suggesting *Tm*XdhA could be xylan targeting and is thus a good candidate for xylooligosaccharide targeting applications. The structure prediction suggests that Q406 promotes the binding between *Tm*XdhA and the xylo-pyranosyl reducing end moiety.

## Methods

### Materials

All buffering chemicals and substrates including glucose, xylose, mannose, galactose, cellobiose, lactose, mannobiose, β-(1 → 4)-_D_-galactobiose, isomaltose and maltose were purchased from Sigma-Aldrich (Germany). The other substrates including cellotriose, cellotetraose, xylobiose, xylotriose, xylotetraose, 3^2^-α-l-arabinofuranosyl-xylobiose (A^3^X), 2^3^-α-l-arabinofuranosyl-xylotriose (A^2^XX), 2^2^-(4-*O*-methyl-α-d-glucuronyl)-xylobiose (U^4m2^X), 2^3^-(4-*O*-methyl-α-d-glucuronyl)-xylotriose (U^4m2^XX), chitosanbiose, 3^2^-β-d-glucosyl-cellobiose, β-(1 → 4)-_D_-glucosyl-_D_-mannose, gentiobiose, kojibiose, sophorose, laminaribiose, and nigerose were purchased from Megazyme (Ireland). The laccase from *T. versicolor* (38429, Sigma-Aldrich, Germany) was used in oxidation reactions to recycle the electron acceptor, 1,4-benzoquinone (BQ, PHR1028, Sigma-Aldrich, Germany). Methylated cellobiose and lactose was produced in house (details in Additional File 2).

### Phylogenetic analysis

To detect proteins of *T. myriococcoides* CBS 389.93 from the Auxiliary Activity families of interest, an hmm-scan search with default cut-off values (if alignment > 80aa, use *E*-value < 1e−5, otherwise use E-value < 1e−3; covered fraction of HMM > 0.3) were performed against all proteins of this organism using HMMs from dbCANv9 database. The protein sequences were retrieved from the Centre of Structural and Functional Genomics at Concordia University [18]. The result from hmm-scan was combined with the best hits from blastp search against all sequences from CAZy database [39] (Additional file 3).

To build the phylogenetic tree of AA3_1 proteins, 206 protein sequences from this subfamily were retrieved from CAZy. Among those, 14 were reported as experimentally characterized. Four AA3_1 proteins of *T. myriococcoides* CBS 389.93 found from the hmm-scan and blastp searches were added to this AA3_1 dataset. The multiple sequence alignment profile was created using MUSCLE [40] and the trimming was described by [13]. CBM1 and cytochrome domain information marked on the tree were obtained from the pfam scan against Pfam database v34. The tree was then constructed and edited using FastTree [41] and iTOL [42].

### RNA extraction, cloning of *xdhA* and recombinant protein production

*Tm*XdhA coding DNA sequence was obtained from the *T. myriococcoides* CBS 398.93 genome portal [18, 43]. The strain was grown on YpSs agar medium for three days at 45 °C at a shaking speed of 220 rpm [44]. Ten plugs from the mycelium grown on agar were used to inoculate a primary culture of 50 ml liquid mycological broth (MB) (1% soytone, 0.4% D-glucose, trace elements, pH 5). *Trametes* defined medium (TDM) containing 2% of a mix of alfalfa and barley was used for the main culture with an inoculum of 10% volume from the primary culture [44]. Mycelia was harvested after 24 h of incubation at 45 °C at a shaking speed of 220 rpm and was grinded into powder as described by [44, 45]. Total RNA was extracted using the RNeasy® Plant Maxi Kit (Qiagen) and complementary DNA (cDNA) was synthesized using the Superscript™ III reverse transcriptase (Invitrogen) according to instructions from manufacturers.

For cloning, *TmxdhA* gene was amplified from cDNA by PCR using Phusion® High-Fidelity DNA Polymerase (New England BioLabs Inc.) The following forward and reverse primers were used: 5ʹ-CCCCAGCAACAAAACACCGGCTCAGCAATGCAAACTGCTTCGAAATTAGC-3ʹ and 5ʹ- GAAGGACGGCGACGGACGGCTTCACGATTCCGCATCCTC-3ʹ. Ligation-independent cloning (LIC) method [46] was used to clone the gene into the LIC-adapted vector pGBFIN49, in which the gene is flanked by 1972 bp of the *Aspergillus niger* glucoamylase A (*glaA*) promoter and 701 bp of the *A. nidulans trpC* terminator.

CRISPR-Cas9 technology was used to replace the *glaA* gene with the *TmxdhA* gene into the genome of the engineered *A. niger* strain CSFG_9057 (FGSC #A1513 *∆pyrG ∆kusA ∆[prtT amyC agdA] ∆bglA ∆laeA ΔglaATt::trpCTt*). Geneious software was used to select the 20-bp guide RNA sequence targeting the *glaA* gene. The guide RNA sequence was cloned into ANEp8-Cas9 as described by [47]. The expression vector containing the *TmxdhA* gene was co-transformed into CSFG_9057 strain with the ANEp8-Cas9 plasmid containing the *glaA*-targeting guide RNA sequence using protoplast-mediated transformation method [48]. Transformants were selected on minimal medium without uracil and uridine [49]. Supernatants from transformants were screened for recombinant protein production after growth in MMJ medium supplemented with 0.1% arginine and containing 15 g/l of maltose for induction of protein production [50].

Spores from transformant producing the *Tm*XdhA recombinant protein were inoculated in 500 ml MMJ medium supplemented 0.1% arginine at a concentration of 2 × 10^6^ conidia/ml. Supernatant was harvested after six days of stationary incubation at 30 °C. Desalting and concentration of the supernatant was done using Vivaflow^®^ cassette as described in the manufacturer’s protocol (Sartorius). Protein production and concentration were checked on SDS-PAGE gel using standard techniques [51].

### Purification of the recombinant protein

The secreted recombinant protein was first concentrated to smaller volume using centrifuge filter with a cut-off of 10 kDa. Afterwards, the concentrated fraction was filtrated through 0.45 µm filter and purified with Size Exclusion Chromatography (SEC, HiLoad 16/600 Superdex 200 pg column, GE healthcare, USA) in 10 mM sodium citrate buffer (pH 5) with 0.15 M sodium chloride. The protein purity was checked with SDS-PAGE for each fraction. Semi-purified fractions of *Tm*XdhA were pooled and loaded to the SEC again for a second round of purification. After that, the purified fractions of *Tm*XdhA were pooled and exchanged to 10 mM sodium acetate buffer (pH 5) and concentrated using 30-kDA cut-off Vivaspin 20 spin columns (Sartorius, Germany). The final protein concentration was measured using the Bradford method (Bio-Rad Laboratories, USA) and the purified protein was snap-frozen and stored in -80 °C in aliquots.

### Confirmation of protein purity by deglycosylation, 2-D electrophoresis and MALDI-TOF-MS

The purified protein was treated with PNGaseF (New England Biolabs, USA) at both denaturing and native conditions at 37 °C for 4 h. The samples before and after deglycosylation at both denaturing and native conditions were analyzed by SDS-PAGE, the specific activity of the purified enzyme that was deglycosylated under native condition was also analyzed to evaluate the impact of glycosylation. Subsequently, the purified enzyme was analyzed by 2-D electrophoresis using pH 3–10 strip and Criterion TGX- 4–20% gel (Bio-Rad Laboratories, USA). The spots from 2D gel were cut, destained, digested with trypsin, and then subjected to MALDI-TOF-MS. Proteins were identified by correlating the mass spectra to the *A. niger* protein database from Uniprot and the *TmxdhA* sequence.

### Enzymatic assays

To select the optimal pH for *Tm*XdhA oxidation reactions, the activity of *Tm*XdhA was screened at 30 °C with 5 mM cellobiose and 1 mM 2,6-Dichloroindophenol (DCIP, D1878, Sigma-Aldrich) in McIlvaine’s buffer at pH values from 3.0 to 7.5. The activity was also followed at temperature ranging from 10 to 70 °C with 5 mM cellobiose and DCIP BQ in 50 mM ammonium acetate buffer (pH 5.5) to determine effect of temperature on *Tm*XdhA activity. The thermostability of *Tm*XdhA was determined by measuring its residue activity at 30 °C in 50 mM ammonium acetate buffer (pH 5.5) after incubation at different temperatures (40, 50, 60 °C) in a concentration of 50 µg/ml for up to 48 h.

The activity of *Tm*XdhA was also analyzed at 30 °C with 5 mM cellobiose and 1 mM BQ in 50 mM ammonium acetate buffer (pH 5.5) to investigate the influence of volatile buffer and e-acceptor. Reduction of DCIP (ε_520_ = 7.8 mM^−1^ cm^−1^) and BQ (ε_290_ = 2.24 mM^−1^ cm^−1^) was followed using an Eon plate reader (BioTek, USA). All reactions were performed in triplicates. The oxidase activity of *Tm*XdhA was measured by following the formation of hydrogen peroxide in a coupled reaction between horseradish peroxidase and ABTS (ε_420_ = 36 mM^−1^ cm^−1^) [52].

Spectrophotometric assays to determine the initial activity were carried out towards glucose and the di- and oligosaccharides that were oxidized after 24 h incubation. Reactions (250 µl) were performed at 30 °C in 50 mM ammonium acetate buffer (pH 5.5) with 5 mM substrate and 1 mM BQ, and the reduction of the BQ was measured for up to 40 min.

Steady-state kinetic constants for cellobiose, cellotriose, cellotetraose, xylobiose, xylotriose, and xylotetraose were measured using the conditions for initial activity determination. The reduction rate of BQ was plotted versus substrate concentration (8 points, from 0 to 5 mM). The Michaelis–Menten constant (*K*_M_) was estimated by fitting of the data to the Michaelis–Menten equation using GraphPad Prism 6.0 (GraphPad Software, USA). The measurements were followed using an Eon plate reader (BioTek, USA). All reactions were performed in triplicates.

The hydrolytic activity was tested on five different *p*NP-sugars. Each reaction mixture contained 2.5 mM substrate in 25 mM ammonium acetate buffer (pH 5.5). 1 µg of *Tm*XdhA was added in each reaction. Reactions were terminated after 20 min incubation at 30 °C by adding equal volume of 1 M Na_2_CO_3_. The activity was determined by measuring *p*NP release at 405 nm and calculated with *p*NP standards.

Laccase activity was measured using 5 mM hydroquinone (HQ, H9003, Sigma-Aldrich, Germany), and oxidation of HQ (ε_249_ = 17.25 mM^−1^ cm^−1^) in 250 μl reaction was followed.

### Substrate screening and identification of reaction products with ESI-Q-TOF-MS

The substrate specificity of *Tm*XdhA was determined by using 29 different carbohydrates (Table 2) including monosaccharides, glucosidic-disaccharides with different glucosidic linkages, β-(1 → 4)-linked disaccharides, cello-/xylo-oligosaccharides, and C-1 methylated cellobiose and lactose. All reactions (125 µL total reaction volume) were performed at 30 °C with shaking (400 rpm) in 10 mM ammonium acetate buffer (pH 5.5) containing 25 mU *Tm*XdhA, 5 mM sugar substrate, 1 mM BQ as e-acceptor, and 25 mU T*.versicolor* laccase for the regeneration of BQ. For the reactions with α-glucosidic-disaccharides, 0.1 mM castanospermine (532,673, Sigma-Aldrich, Germany) was included to inhibit the hydrolytic side activity [53]. Oxygen availability was not controlled during the reactions. The sampling (50 µL) was done at 3 h for the reaction mixtures containing A^3^X and A^2^XX and at 24 h for all other reactions.

Reactions were stopped after sampling by filter through 10-kDa cut-off Vivaspin 500 spin columns (Sartorius, Germany). Mass spectrometric analysis was then done for checking if the substrates were oxidized by *Tm*XdhA and to identify the reaction products using Quadruple Time-of-flight (Q-TOF) mass spectrometry with an ESI source (SYNAPT G2-Si, Waters, MA, USA). In practice, a 5 μl sample from each reaction was mixed with 5 μl of 10 mg/ml ammonium chloride and 500 μl 50% methanol in water prior introducing to the ESI-Q-TOF-MS. The analysis was done in negative mode and the ions were collected in *m/z* range of 50 to 1200 with the parameters developed by [54]. Fragmentation analysis was done to ions generated from oxidized products to identify the reaction products.

### Enzymatic conversion of cellooligosaccharide and xylooligosaccharide series

The efficiency of *Tm*XdhA for oxidizing cello- and xylo-oligosaccharides was compared individually and in mixtures. The reactions (250 µL) were performed at 30 °C in 10 mM ammonium acetate buffer (pH 5.5) with shaking (400 rpm) using 0.2 mM BQ as an e-acceptor in triplicates. For the reactions on each substrate individually, 1.25 mU *Tm*XdhA and 3.75 mU *T. versicolor* laccase were added to convert 5 mM substrate. For the conversions of oligosaccharide mixtures, the enzyme dose to molarity of reducing end ratio was kept the same, with 3.75 mU of *Tm*XdhA and 11.25 mU of laccase added to convert a mixture of three oligosaccharides in equal molarity (5 mM each). Five series were studied, including cellooligosaccharide series with cellobiose, cellotriose, and cellotetraose and xylooligosaccharide series with xylobiose, xylotriose, and xylotetraose. The three other series mixed cello- and xylo-oligosaccharides with combinations of xylotriose, xylotetraose, and cellotetraose; xylotriose, cellotriose, and cellotetraose; and xylobiose, cellobiose, and xylotetraose.

Time course sampling (50 µL) was done at 1, 3, 7, and 24 h. The oxygen level was not controlled. Reactions were stopped by adding 200 µL 0.1 M ammonia solution directly after sampling and filtering through 10 kDa centrifuge filter. The flow through were lyophilized and redissolved in 50% ACN. The samples were kept frozen at − 80 °C prior to further analysis by mass spectrometry and HILIC-ELSD.

### Semi-quantitative analysis by mass spectra

5 μL sample solution from each time point was mixed with 5 μl of 10 mg/ml ammonium chloride and 500 μl 50% methanol in water before introducing to ESI-Q-TOF-MS. Ions were analyzed in negative mode and collected in *m/z* range of 50 to 1200. The oxidized substrates were deprotonized and the original substrates presented as chloride adducts. The following equation was used d to estimate the extent of oxidation completeness employing the relative ratio in peak height between the non-oxidized substrate and the formed product:

1 $$\mathrm{Oxidation completeness}=\frac{\mathrm{Abundance of isotopic}\, m/z \,\mathrm{for oxidized substrate}}{\mathrm{Abundance of isotopic }\,m/z\,\mathrm{ for oxidized subatrate }+\mathrm{ abundance of isotopic }\,m/z\,\mathrm{ for original substrate}}.$$

### Quantification of oxidized products by HILIC-ELSD

The depletion of substrates and formation of the corresponding aldonic acids were followed using an Acquity UPLC coupled with evaporative light scattering detector (ELSD). A 1.7 μm, 2.1*150 mm Acquity UPLC BEH Amide column (HILIC amide, Waters, MA, USA) was used to separate the reaction products. The elution gradient and instrument settings were according to [54]. External standard series with cello- and xylo-oligosaccharides, and their corresponding aldonic acids were made by injecting 200 ng to 1500 ng of each compound. The injection volume for the samples varied so that the injection amount of each substrate and its reactions product fall in the quantification range. Each standard curve was fitted to a quadratic polynomial equation of f(*x*) = a*x*^2^ + b*x* + c, where f(*x*) is the peak area, *x* is the sample amount.

The aldonic acids were made in-house using wild-type glucooligosaccharide oxidase (GOOX) from *S. strictum* [36]. The GOOX activity was determined according to [55]. For aldonic acids production, 13.5 mg/mL substrate was oxidized for 24 h at 37 °C in 10 mM ammonium acetate buffer (pH 5.5) by 400 mU/mL GOOX. Catalase from bovine liver (1200 mU/mL, C30, Sigma, Germany) was also included to remove the formed hydrogen peroxide. The reaction mixtures after 24-h incubation were filtered through 10 kDA centrifugal filters and lyophilized to dryness to recover the aldonic acids. The pure aldonic acids were redissolved to proper dilution series with 50% ACN prior HILIC-ELSD injection.

### Sequence alignment and structure prediction

The multiple sequence alignment with *Tm*XdhA, cellobiose dehydrogenase from *N. crassa* (PDB code 4QI7), and *Mt*CDH cellobiose dehydrogenase from *T. myriococcoides* (PDB code 4QI4, strain CBS 208.89) was created using MUSCLE [40]. A structural model of *Tm*XdhA was built using a colab version of AlphaFold2 [26] and the model was displayed with FAD cofactor added using PyMOL v 2.1.0 (PyMOL Molecular Graphics Systems, Schrödinger, LLC). The PyMOL molecular graphic system (V 2.1.0, Schrödinger, LLC) was used for structure visualization and structural alignments.

### Statistical analyses

Averages and standard deviations were calculated over three replicate reactions (*n* = 3). One-way ANOVA (*p* < 0.05; Graphpad) with *F*-test and Tukey’s test were performed to ascertain the difference in specific activities towards different substrates and *K*_M_ values.

## Supplementary Information

**Additional file 1: **Details of the characterized AA3_1 proteins in CAZy database.

**Additional file 2: Table S1.** The summary of the activity of *Tm*XdhA for hydrolysing selected nitrophenyl-sugars. **Fig. S1.** (A) SDS-PAGE of purified native *Tm*XdhA (B) 2D electrophoresis gel separation of the purified *Tm*XdhA protein **Fig. S2.** Negative MS spectra of XdhA oxidized A^3^X and A^2^XX. **Fig. S3.** Negative ion MS spectra of purified me-lactose. **Fig. S4.** Negative ion MS spectra of purified me-lactose. **Fig. S5.** Product formation and substrate depletion for mixture reactions by *Tm*XdhA followed by HILIC-ELSD. **Fig. S6.** Per-residue confidence (pLDDT) score and the predicted aligned error (PAE) for *Tm*XdhA model generated with Alphafold2. Note 1: Methylation of cellobiose and lactose. Note 2: Thin-layer chromatography for SEC purified methylated lactose and methylated cellobiose.

**Additional file 3: **Multiple sequence alignment profile for the dehydrogenase domain of AA3_1 proteins.

## Data Availability

The datasets generated for this study are available on request to the corresponding author.
