# Supplementary material for: Characterization of a novel AA3_1 xylooligosaccharide dehydrogenase from Thermothelomyces myriococcoides CBS 398.93
Source: Biotechnol Biofuels Bioprod. 2022 Dec 7;15:135. doi: 10.1186/s13068-022-02231-w (PMC9730589; doi:10.1186/s13068-022-02231-w)
Supplement: Supplementary file 2 — Additional file 2: Table S1. The summary of the activity of TmXdhA for hydrolysing selected nitrophenyl-sugars. Fig. S1. (A) SDS-PAGE of purified native TmXdhA (B) 2D electrophoresis gel separation of the purified TmXdhA protein Fig. S2. Negative MS spectra of XdhA oxidized A3X and A2XX. Fig. S3. Negative ion MS spectra of purified me-lactose. Fig. S4. Negative ion MS spectra of purified me-lactose. Fig. S5. Product formation and substrate depletion for mixture reactions by TmXdhA followed by HILIC-ELSD. Fig. S6. Per-residue confidence (pLDDT) score and the predicted aligned error (PAE) for TmXdhA model generated with Alphafold2. Note 1: Methylation of cellobiose and lactose. Note 2: Thin-layer chromatography for SEC purified methylated lactose and methylated cellobiose. [file 13068_2022_2231_MOESM2_ESM.docx]

**Additional File 1**

**Characterization of a novel AA3_1 xylooligosaccharide dehydrogenase from *Thermothelomyces myriococcoides***

Table S1. The summary of the activity of *Tm*XdhA for hydrolysing selected nitrophenyl-sugars with/without addition of 0.1 mM castanospermine.

| **Substrate** | **Activity (U/mg)** | |
| --- | --- | --- |
|  | - castanospermine | +castanospermine |
| 4-Nitrophenyl-alpha-D-glucopyranoside | 0.10±0.03 | n.d. |
| 4-Nitrophenyl-beta-D-glucopyranoside | n.d. | n.d. |
| 4-Nitrophenyl-alpha-L-arabonofuranoside | 0.52±0.03 | 0.50±0.04 |
| 4-Nitrophenyl-beta-D-mannopyranoside | n.d. | n.d. |
| 4-Nitrophenyl-beta-D-xylopyranoside | n.d. | n.d. |

*n.d., not detected

Figure S1. **A**) SDS-PAGE of purified native *Tm*XdhA (lane 1), deglycosylated *Tm*XdhA under denatured condition (lane 2), and deglycosylated *Tm*XdhA under native condition (lane 3). Around 40% loss in *Tm*XdhA molecular weight and 46% decrease in enzyme activity was detected after deglycosylation by PNGaseF. **B**) 2D electrophoresis gel separation of the purified *Tm*XdhA protein (pH 3-10 strip, Criterion TGX+gel 4-20%). Spot 1: *A. niger* glycoside hydrolase (Uniport: A0A370CB09) Spot 2: *Tm*XdhA Small dot with round Spot 3: contaminants from staining


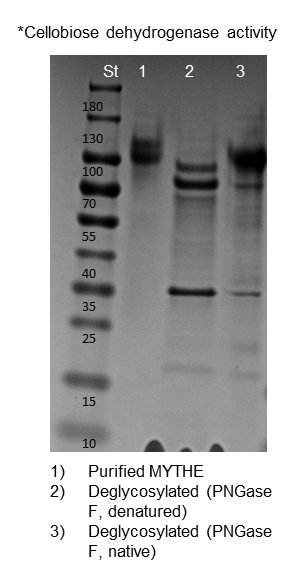


A

B

3.

3.


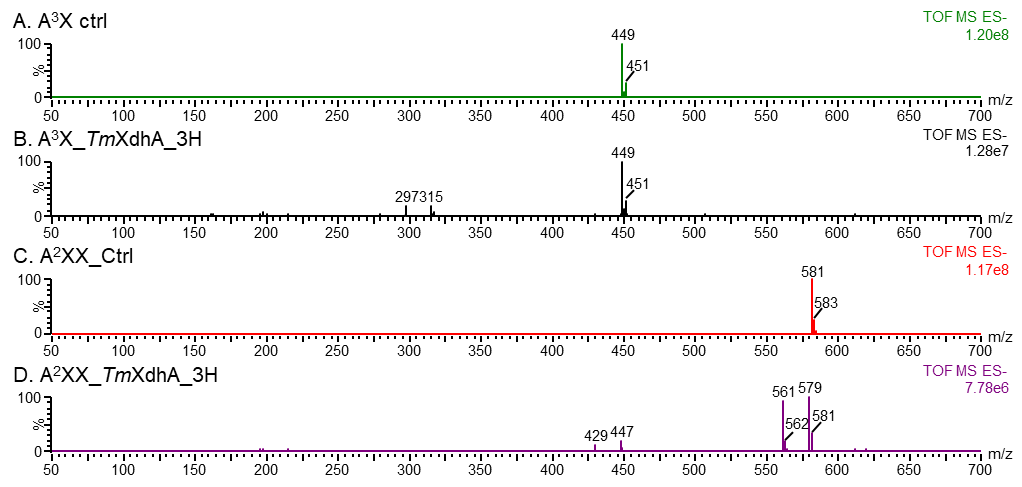


Figure S2. Negative MS spectra of *Tm*XdhA oxidized 3^2^-α-L-arabinofuranosyl-xylobiose (A^3^X) and 2^3^-α-L-arabinofuranosyl-xylotriose (A^2^XX) after 3h. (A) A^3^X after control reaction. (B) A^3^X after 3h reation with *Tm*XdhA. A^3^X was partially oxidized to xylobiose by the residue arabinofuranosidase activity and further oxidized by *Tm*XdhA to form peaks at *m/z* 315 and *m/z* 297. (C) A^2^XX after control reaction (D) A^2^XX after 3h reaction with *Tm*XdhA. A^2^XX was partially oxidized to xylotriose by the residue arabinofuranosidase activity and further oxidized by *Tm*XdhA to form peaks at *m/z* 447 and *m/z* 429. The A^2^XX itself was also oxidized at reducing end hydroxyls to form carboxylic acid with peaks at *m/z* 579 and *m/z* 561.

Figure S3. Negative ion MS spectra of purified me-lactose. Me-lactose is shown both as chloride adduct with m/z of 391 and deprotonized with m/z of 355.

Figure S4. Negative ion MS spectra of purified me-cellobiose. Me-cellobiose is shown both as chloride adduct with m/z of 391 and deprotonized with m/z of 355.



Figure S5. Product formation and substrate depletion for mixture reactions by *Tm*XdhA followed by HILIC-ELSD. Cellobiose, C2; cellotriose, C3; cellotetraose, C4; xylobiose, X2; xylotriose, X3; xylotetraose, X4.


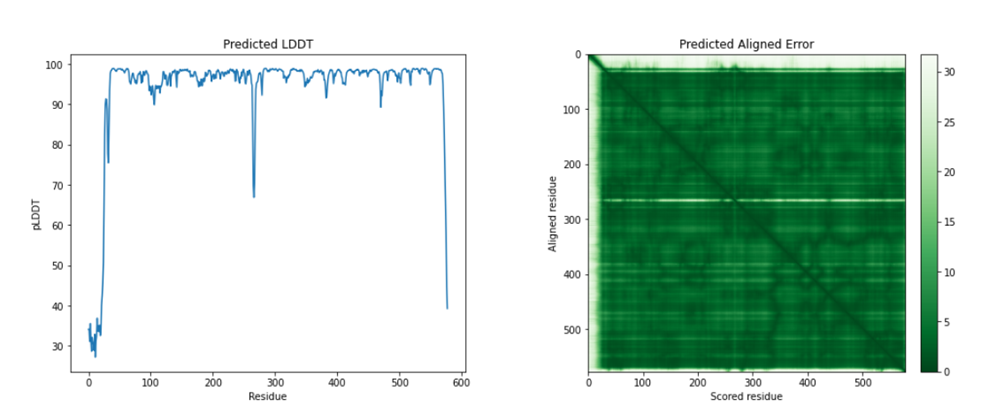


Figure S6. Per-residue confidence (pLDDT) score and the predicted aligned error (PAE) for TmXdhA model generated with Alphafold2.

**Note 1: Methylation of cellobiose and lactose**

Cellobiose and lactose were methylated at the free anomeric carbon to block the reducing end. The methylation conditions for both cellobiose and lactose were optimized for the highest purity and yield. Specifically, cellobiose and lactose were dissolved in 1 M HCl in methanol at a concentration of 1 mg/mL and 5 mg/mL respectively. The mixtures were stirred overnight at room temperature. Subsequently, equal molar amount of NaOH was added in the mixtures to stop the reaction. The supernatant was collected after centrifugation at 8,000 g for 5 minutes, evaporated to dryness with a rotate evaporator, and redissolved in water. The water solution was filtered with 0.45 µm nylon syringe filter (Pall Corp., Ann Arbor, MI, USA) and then loaded to a size exclusion column packed with Bio-Gel P-2 fine gel (1.5 × 70 cm, Bio-Rad Laboratories, CA, USA) for desalting and purification. The column matrix was equilibrated and washed with 20 mM ammonium acetate buffer (pH 7) before the sample loading. The products were fractionated and eluted with 20 mM ammonium acetate buffer (pH 7) at a flow rate of 0.3 mL/min. Conductivity was monitored to track the elution of salt and Thin-layer chromatography was carried out to locate the products. The pure fractions were then pooled together; the purity was further checked by direct infusion ESI-Q-TOF before being lyophilized to dryness. The powder was then dissolved in concentration of 20 mg/mL and stored in -20 °C for further analysis and use.

**Note 2: Thin-layer chromatography for SEC purified methylated lactose and methylated cellobiose**

10 µl sample from each fraction was spot onto TLC silica gel 60 plate (Merck, Germany) and stained with orcinol reagent (80% EtOH v/v, 10% H_2_SO_4_ v/v, 10% H_2_O v/v, 0.2% orcinol w/v). The fractions containing carbohydrate were then picked and spotted again onto TLC plate and separate with 1-BuOH: Ethanol: Water (3:2:2) and stain with orcinol reagent. The fractions with pure products were pooled and lyophilized to completely dryness. The final weight was recorded to for the calculation of the yield. The purity is confirmed with mass spectrometry (Figure S3 & 4). The recovery rates for me-lactose and me-cellobiose were 41.8% and 51.4% respectively.
